# Pre-Emergency-Department Care-Seeking Patterns Are Associated with the Severity of Presenting Condition for Emergency Department Visit and Subsequent Adverse Events: A Timeframe Episode Analysis

**DOI:** 10.1371/journal.pone.0127793

**Published:** 2015-06-01

**Authors:** Chien-Lung Chan, Wender Lin, Nan-Ping Yang, K. Robert Lai, Hsin-Tsung Huang

**Affiliations:** 1 Department of Information Management and Innovation Center for Big Data and Digital Convergence, Yuan Ze University, Chung-Li, Taiwan; 2 Department of Health Care Administration, Chang Jung Christian University, Tainan, Taiwan; 3 Community Health Research Center & Institute of Public Health, National Yang-Ming University, Taipei, Taiwan; 4 Department of Computer Science and Engineering, and Innovation Center for Big Data and Digital Convergence, Yuan Ze University, Chung-Li, Taiwan; 5 Medical Affairs Division, National Health Insurance Administration, Ministry of Health and Welfare, Taipei, Taiwan; 6 Keelung Hospital, Ministry of Health and Welfare, Keelung, Taiwan; Azienda Ospedaliero-Universitaria Careggi, ITALY

## Abstract

**Background:**

Many patients treated in Emergency Department (ED) visits can be treated at primary or urgent care sectors, despite the fact that a number of ED visitors seek other forms of care prior to an ED visit. However, little is known regarding how the pre-ED activity episodes affect ED visits.

**Objectives:**

We investigated whether care-seeking patterns involve the use of health care services of various types prior to ED visits and examined the associations of these patterns with the severity of the presenting condition for the ED visit (EDVS) and subsequent events.

**Methods:**

This retrospective observational study used administrative data on beneficiaries of the universal health care insurance program in Taiwan. The service type, treatment capacity, and relative diagnosis were used to classify pre-ED visits into 8 care types. Frequent pattern analysis was used to identify sequential care-seeking patterns and to classify 667,183 eligible pre-ED episodes into patterns. Generalized linear models were developed using generalized estimating equations to examine the associations of these patterns with EDVS and subsequent events.

**Results:**

The results revealed 17 care-seeking patterns. The EDVS and likelihood of subsequent events significantly differed among patterns. The ED severity index of patterns differ from patterns seeking directly ED care (coefficients ranged from -0.05 to 0.13), and the odds-ratios for the likelihood of subsequent ED visits and hospitalization ranged from 1.18 to 1.86 and 1.16 to 2.84, respectively.

**Conclusions:**

The pre-ED care-seeking patterns differ in severity of presenting condition and subsequent events that may represent different causes of ED visit. Future health policy maker may adopt different intervention strategies for targeted population to reduce unnecessary ED visit effectively.

## Introduction

The growing patient volume in over-crowded emergency departments (EDs) has posed constant challenges for health care systems worldwide. Among possible contributing factors to this problem, ED use by considerable amount of patients with nonurgent conditions is a primary concern for researchers and policy makers. Most previous studies hypothesized that ED use is associated with the characteristics of both patients and their primary care systems [1–8]. Patients seeking ED care tend to have unmet health care needs resulting from limited or untimely access to alternative sources of care. Based on these findings, improving accessibility to primary care and alternative services has become a key strategy for reducing ED visitation.

However, intervention programs have yielded mixed results [8–11]. A previous study observed reduced ED use after the increase of accessibility was increased [9], however, another study reported that ED care seeking behaviors remained unchanged, even when accessibility was improved [12]. Numerous nonurgent ED visits occurred in areas where primary care services were highly available [13,14] and providing additional primary care did not replace visits to ED [15,16]. In addition, studies have indicated that ED users received a substantial amount of health care [17–19], and a large proportion of ED visitors sought other type of care prior to ED visits [15,20,21]. The variation in these results indicates that it is merely increasing the availability of alternative sources of care is an insufficient strategy for reducing ED use. The characteristics of patients seeking ED care result from complex dynamics of interaction between health care demand and supply [22]. Therefore, understanding the various pre-ED care-seeking activities initiated by patients before they visit an ED is crucial.

Previous studies have reported that a substantial proportion of ED patients could have been treated at primary or urgent care sectors [23–25], despite a large proportion of ED visitors have sought other care prior to ED. However, most previous studies examining pre-ED care-seeking behaviors have used either questionnaires to survey medical contact status [15,20,26,27] or quantization to observe patients’ care use before visiting an ED [14,17,18,28,29]. Because those studies used data based on patient self-report or simple quantitative analysis for care usage in a relative long days before ED visit, the results may not reveal sufficient information regarding what actually occurred prior to ED visit. This study was conducted to determine and confirm the associations among pre-ED patient-initiated care-seeking patterns based on various care types, the severity of presenting condition for ED visit (EDVS), and subsequent event.

## Methods

### Research Setting

Taiwan’s national health insurance (NHI) program provides a comprehensive and unified health care benefit package to Taiwanese citizens [30]. In 2010, approximately 99% of the 23 million Taiwanese citizens were enrolled in the NHI program, and over 92% of all medical facilities, including all hospital-based facilities, were contracted with the NHI program [13]. In this program, beneficiaries can choose to receive care at any contracted facility, and access to physicians is not limited by referral mechanism. Therefore, in Taiwan, hospital-based ED care is an option for patients with nonurgent conditions and, thus, may be inappropriately used [1,13]. The NHI full-population database consists of numerous complete claims data that have been analyzed by researchers from multiple-disciplines. Thus, Taiwan is an ideal setting for studying the care-seeking behaviors in which patients engage before visiting an ED.

### Data Source

Data were obtained from the Longitudinal Health Insurance Database 2005 (LHID2005), and the registry for beneficiaries and contracted medical facilities, all of which are stored in the National Health Insurance Research Database (NHIRD) and is managed by Taiwan National Health Research Institute for policy and research analysis. The NHIRD comprises a group of databases containing NHI administration and claim data. The LHID2005 contains medical use information on 1 million randomly selected beneficiaries who were enrolled in the NHI program in 2005. Information on every beneficiary collected between 1996 and a recent year can be analyzed. The research data in this study has undergone data encryption and privacy protection by IT department of NHIA (National Health Insurance Administration) prior analysis, and permission to use the data has been approved by an administration approval process in NHIA in Taiwan.

**Fig 1:**
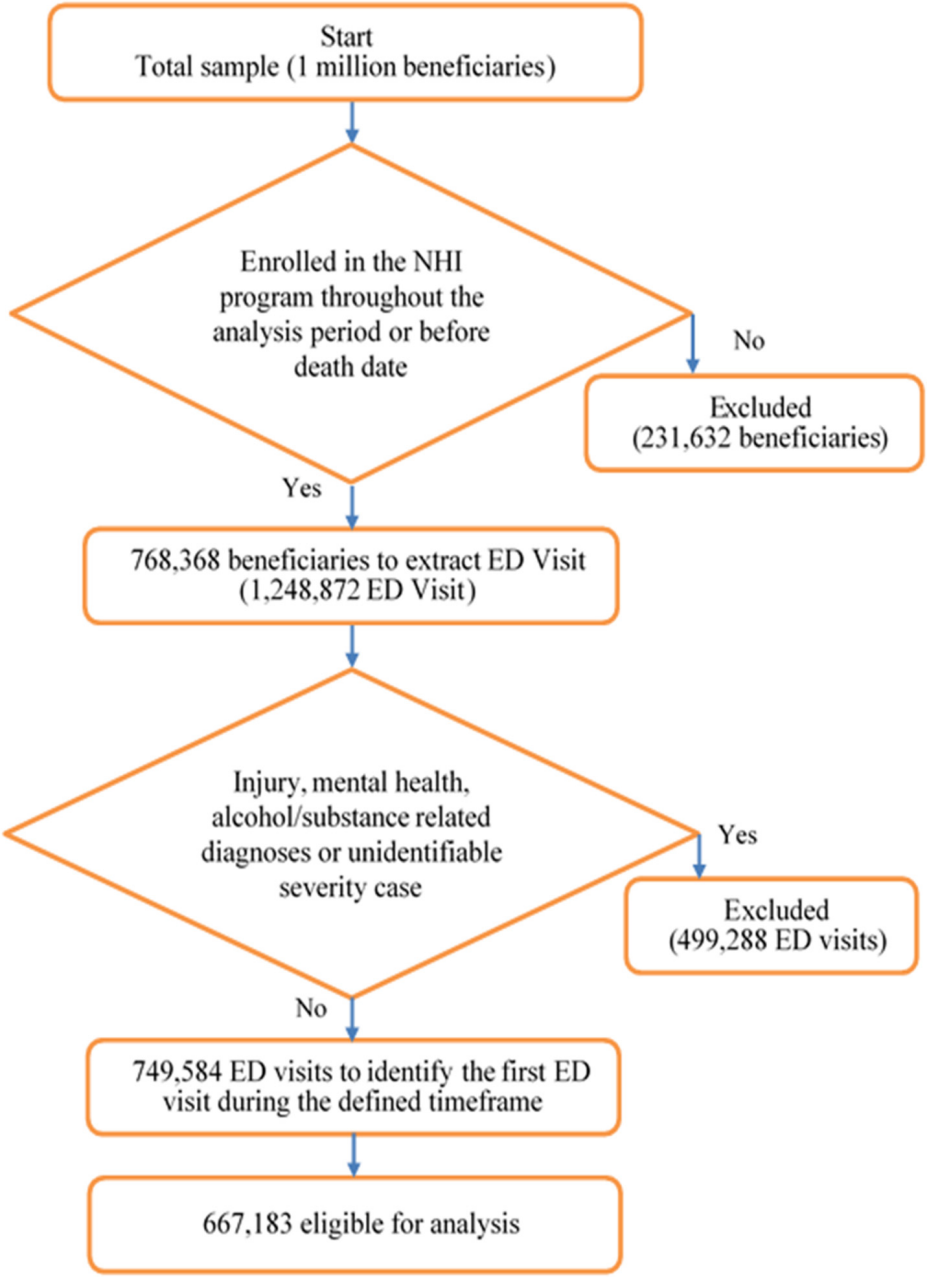
This is the Fig 1 The flow chart of data process.

Records of ED visits that occurred during 2005 to 2010 were retrieved from the LHID2005 and were defined as index ED visit. To ensure that the patient information was intact, data on beneficiaries who had not been enrolled in the NHI program throughout the analysis period were excluded; however, which of the beneficiaries who had died during 2005–2010 and fully enrolled in the NHI program before death date were not excluded. To increase the homogeneity of the study patients and to focus on disease-related ED visits, ED visits related to the following conditions or diagnoses were excluded: injuries, alcohol/substance abuse, treatments for mental illness, or disease of unidentifiable severity. The flow of data process showed in Fig 1. Data on the use of all non-ED medical services, except dental care and traditional Chinese medicine, before and after index ED visits were retrieved.

### Setting Episode Timeframes

The analysis unit in this retrospective observational study was the concurrent timeframe episode which is defined as care-seeking activities prior to an index ED visit initiated by patients. The timeframe can vary widely, and studies have used various time intervals [31–33]. Certain studies have employed an algorithm that groups related care service use based on health insurance billing codes, within 3 days before an ED visit [28,34], others study on patient flows during the week prior to an ED visit [35]. Assuming that a patient’s need for service may arise before he or she visits an ED, we set a timeframe of 7 days to observe pre-ED care-seeking behaviors and a timeframe of 7 days after ED discharge to observe subsequent medical events including the ambulatory visits occurred on the same day of index ED visits. Finally, 667,183 ED visits that were first ED visit during the defined timeframe were eligible for analysis of pre-ED timeframe episode.

### Defining and Patterning Pre-Emergency-Department Care-Seeking Episodes

We identified and patterned the episodes by using a 3-step process. First, every pre-ED health care visit was assigned to one of 8 care types based on the following 3 topics issues: care services, diagnosis relationship, and treatment capacity. Care services were classified into 3 types, namely no health care visit (NHC), hospitalization (HP), and ambulatory care. NHC, a dummy for care-seeking, indicates that the patient directly sought ED care. Study have considered HP to be an adverse outcome of previous medical services [36]. Ambulatory care can represent various reasons to visit an ED and can be divided further into 6 care types based on whether (1) the diagnosis is related to an ED visit; and (2) whether the visit occurred in the same facility at which the ED visit was conducted or in a facility at which the level of treatment complexity is higher, lower than or equal to that at which the ED was visited. Based on a diagnosis use the ICD-9-CM code group that 3-digit category codes, if any one of the 3 diagnoses recorded in the claims data on the pre-ED visit matched any one of the 3 diagnoses of index ED visit, then the visit were classified as involving a disease-related to the ED visit; all other visits were considered as involving nonrelated-disease.

Second, to extract the sequential patterns of frequent pre-ED care-seeking, we used frequent sequential pattern (FSP) analysis based on the method of association rule mining, which has been widely used in data mining to support frequent pattern analysis [37]. Consequently, 17 sequential care-seeking patterns were extracted when setting 1% minimal support and a maximum number of visits of 3 were set as the criteria.

Finally, we identified a single sequential care-seeking pattern for every eligible pre-ED episode. For multi-patterns episodes, the final care-seeking pattern was determined according to the following sequent criteria in consideration of the information sufficiency and relationship representativeness of the pattern: (1) no other super-pattern exists in the episode timeframe (ie, maximal frequent pattern); (2) the final visit in the pattern was related the ED visit; (3) the time between the final visit and the ED visit is the shortest.

In addition, to distinctly indicate that patients visit an ED directly after a related pre-ED visit type, the 17 extracted patterns can be classified into 3 groups with 8 subgroups based on the final visit type in the pre-ED care-seeking action.

### Outcome Measures

The primary outcome variables were the severity of the presenting condition for the ED visit (EDVS) and events after ED discharge. We determined the EDVS by using the New York University (NYU) ED classification algorithm (EDCA) developed by The NYU Center for Health and Public Service Research [38]. Based on the principal ICD-9-CM diagnosis code, the EDCA assigns probabilities in 4 severity categories: Nonemergent (NE); Emergent/Primary Care Treatable (EPCT); ED Care Needed, Preventable/Avoidable (EDCNPA); and ED Care Needed, not Avoidable (EDCNNPA). We used the percentage of combined probabilities of the 2 highest severity categories (EDCNPA and EDCNNPA); this percentage is referred to as the EDVS index. The value of the EDVS index ranges between 0 and 100, and a higher value indicates the ED visit has a higher likelihood of being an emergency visit. For the events following ED discharge, we assumed that if ED visit were truly high severity, they would be followed by relatively higher proportions with hospitalization and/or return to an ED [39,40]. Thus, we used claims data to identify two subsequent medical events, namely return to an ED (RED) and hospital admission (HA) to examine the pre-ED care-seeking difference in the events.

### Confounding Variables

Several covariates were shown to influence non-emergency ED visits in our previous studies [13], and were thus considered confounding variables: (1) patient socio-demographic characteristics, including age, sex and beneficiary identities; (2) the Charlson Comorbidity Index (CCI), serving as a proxy for the health status in the year prior to the ED visit. We used the Romano adaptation of Charlson comorbidity index, a summary measure of 17 chronic disease diagnoses, based on ICD-9-CM codes, from administrative data that were selected and weighted according to their association with mortality [41], and calculated each ED visiting patient’s CCI score in the 1 year period prior to the index ED visit; (3) the continuity of care index (COCI), serving as a proxy for overall medical use behavior [42] in the year before the ED visit (when the COCI is applied, a 3-quartile group is used to reflect the extent of the dispersion of outpatient visits); (4) characteristics of the ED visit, such as the levels of the care site and time of the visits (during weekends or holidays). When the association with subsequent events was examined, the EDVS was also considered a confounding variable; an ED visit for a more sever medical condition is associate with a higher likelihood that subsequent adverse events occur [39].

### Data Analysis

We examined the difference in the EDVS and the likelihood of subsequent events among the care-seeking patterns while controlling for variation in the confounding variables. Generalized linear models (GENMODs) were used in this study based on outcome variables to select an appropriate link function and response probability distribution. Because the distribution of EDVS values was skewed right, we applied a gamma distribution with a logarithmic link function. To avoid missing calculation of the logarithmic function, we set the value of the 0.00001 instead of 0 value of EDVS. The likelihood of subsequent events was expressed using binary variables; thus, a binomial distribution with a logit link function was applied. In additional, to resolve the problem of correlated data arising from repeated observations of similar patients, we constructed a GENMOD by using generalized estimating equation (GEE) methods to analyze the association between care-seeking patterns and outcome variables. All data processing, FSP analyses and statistical analyses were performed using SAS Version 9.3 software and SAS Enterprise Mining Workstation 7.1 (SAS Institute Inc., Cary, NC).

## Results

### Characteristics of Patients and Emergency Department Visits

Half of the patients who visited an ED examined in this study were aged between 19 and 64 years. Those above 65 years of age accounted for 25.63%. No difference in sex existed among the patients. Moreover, 42.35% of the patients, primarily those who were employed by specific employers, belonged to Beneficiary Category I. Of the patients, 54.72% a CCI score of 0, and 46.82% had a medium COCI score. In addition, 46.80% of the patients received ED services at a metropolitan hospital; 27.73% received ED services at an academic medical center, and 24.84% received ED services at a local community hospital. Of the ED visits, 41.04% occurred during weekends and holidays (Table 1).

**Table 1:** The characteristics of patients and index ED visits.

	Number of ED visits/episodes	
Items	n	(%)
Total	6,67,183	100.00
Socio-demographic		
Age		
0–18	1,67,819	25.15
19–64	3,28,347	49.21
≧ 65	1,71,017	25.63
Sex		
Male	3,39,192	50.84
Female	3,27,991	49.16
Beneficiary category		
Category I	2,82,521	42.35
Category II	1,14,147	17.11
Category III	1,30,770	19.60
Category IV	2,884	0.43
Category V	14,519	2.18
Category VI	1,22,342	18.34
Health status & behavior		
CCI score		
0	3,65,048	54.72
1–2	1,79,965	26.97
≧ 3	1,22,170	18.31
COCI		
Low	1,62,311	24.33
Medium	3,12,342	46.82
High	1,72,147	25.80
Can not measure	20,383	3.06
Characteristics of ED visit		
Hospital level		
Medical center	1,85,007	27.73
Metropolitan hospitals	3,12,225	46.80
Local community hospitals	1,65,695	24.84
Clinics	4,256	0.64
Weekends & holidays		
No	3,93,385	58.96
Yes	2,73,798	41.04

ED indicates emergency department; CCI indicates Charlson comorbidity index before one year of index ED visit; COCI, contiunity of care index before one year of index ED visit.

### Pre-Emergency Department Health Care Visit

Table 2 shows a total of 632,843 pre-ED health care visits that occurred during the 7-day timeframe prior to the 667,183 ED episodes, of which 44.82% were not preceded by the use of other health care service. The remaining 55.18% of the ED episodes were preceded by HP and/or ambulatory care visits. Of the ED episodes, 29.57% were related to at least one pre-ED visit for a related-disease at a lower- or equal-level facility (RDLF), accounting for 44.45% of the total pre-ED visits, whereas 17.82% of the episodes were related to at least one pre-ED visit for a nonrelated-disease at a lower- or equal-level facility (NDLF), accounting for 26.09% of the total pre-ED visits. Moreover, 8.62% and 7.24% of the episodes were related to at least one pre-ED visit for a related-disease at the facility at which the ED visit occurred (RDSF) and one pre-ED visit for a nonrelated-disease at the facility at which the ED visit occurred (NDSF), respectively. Of the pre-ED visits, 20.15% occurred at the same facility; 10.72% were for a related-disease and 9.43% were for a nonrelated-disease. In addition, 2.39% and 3.11% of the episodes were related to at least one pre-ED visit for a related-disease at a higher-level facility (RDHF) and one pre-ED visit for a nonrelated-disease at a higher-level facility (NDHF), respectively. Furthermore, 6.78% of the pre-ED visits occurred at higher-level facilities.

**Table 2:** Pre-ED health care visit by types.

Abbreviation of care type		Number of pre-ED visit(Total visits = 632,843)		Care type including episodes(Total episodes = 667,183)	
	Description of pre-ED care type	n	(%)	n	Support(%)
Seeking directly ED care					
NHC	No healthcare visit during timeframe period.			2,99,016	44.82
Hospitalization					
HP	Hospitalization.	15,992	02.53	15,249	2.29
Ambulatory care					
RDSF	with related-disease at same facility.	67,815	10.72	57,500	8.62
RDLF	with related-disease at facility of lower/equal level.	2,81,326	44.45	1,97,263	29.57
RDHF	with related-disease at facility of higher level.	18,287	02.89	15,951	2.39
NDSF	with non-related-disease at same facility.	59,693	09.43	48,335	7.24
NDLF	with non-related-disease at facility of lower level.	1,65,104	26.09	1,18,904	17.82
NDHF	with non-related-disease at facility of higher level.	24,626	03.89	20,770	3.11

ED indicates emergency department.

Support indicates the percentage of episode that contain the care type of visit.

### Frequent Patterns with Sequential Care-Seeking

FSP analysis revealed 17 sequential care-seeking patterns (Table 3). Of the episodes, 44.82% were classified as NCH patterns (Pattern-01), 1.27% were classified as HP patterns (Pattern-02), and the remaining 53.91% were classified as ambulatory care with relevant visit types (Patterns-03–17). Most pre-ED care-seeking patterns in ambulatory care were RDLF patterns (25.82%; Patterns-06–09), of which 59.18% comprised a single visit. Of the multiple-visit RDLF patterns, 20.64% comprised 2 consecutive visits, 9.75% comprised 3 visits, and 10.43% were preceded by an NDLF visit. NDLFs accounted for 13.19% of the episodes (Patterns-13–16). Of the NDLF patterns, 57.59% comprised a single visit, 19.69% comprised 2 consecutive visits, 10.55% comprised 3 visits, and 12.16% were preceded by an RDLF visit.

**Table 3:** The pre-ED care-seeking patterns/episodes and distribution.

(Total episodes = 667,183)			
Pattern groups	Number of episodes/patterns		
Care-seeking patterns	n	%	Subgroup rate (%)
Seeking directly ED care			
01: NHC→ED	2,99,016	44.82	100.00
To ED after hospitalization			
02: HP→ED	8,459	1.27	100.00
To ED after ambulatory care			
with RDSF (related-disease at same facility)			
***Subtotal***	***45*,*519***	***6*.*82***	***100*.*00***
03: RDSF→ED	31,098	4.66	68.32
04: RDSF*2→ED	6,864	1.03	15.08
05: RDLF→RDSF→ED	7,557	1.13	16.60
with RDLF (related-disease at facility of lower or equal level)			
***Subtotal***	***1*,*72*,*240***	***25*.*82***	***100*.*00***
06: RDLF→ED	1,01,931	15.28	59.18
07: RDLF*2→ED	35,555	5.33	20.64
08: RDLF*3→ED	16,787	2.52	9.75
09: NDLF→RDLF→ED	17,967	2.69	10.43
with RDHF (related-disease at facility of higher level)			
10: RDHF→ED	9,972	1.49	100.00
with NDSF (non-related-disease at same facility)			
***Subtotal***	***32*,*235***	***4*.*83***	***100*.*00***
11: NDSF→ED	24,243	3.63	75.21
12: NDSF*2→ED	7,992	1.20	24.79
with NDLF (non-related-disease at facility of lower or equal level)			
***Subtotal***	***88*,*010***	***13*.*19***	***100*.*00***
13: NDLF→ED	50,685	7.60	57.59
14: NDLF*2→ED	17,331	2.60	19.69
15: NDLF*3→ED	9,288	1.39	10.55
16: RDLF→NDLF→ED	10,706	1.60	12.16
with NDHF (non-related-disease at facility of higher level)			
17: NDHF→ED	11,732	1.76	100.00

ED indicates emergency department; NHC, no health care visit.

"99: XXXX*^n^" indicates the pattern 99 comprised n times consecutive visit of XXXX care type.

RDSF patterns (Patterns-03–05) accounted for 6.82% of the episodes, of which 15.08% were related to 2 consecutive visits and 16.60% were preceded by an RDLF visit. NDSF patterns (Patterns-11–12) accounted for 4.83% of the episodes, of which 24.79% were preceded by 2 consecutive RDSF visits. RDHF and NDHF patterns accounted for 1.49% (Pattern-10) and 1.76% (Pattern-17) of the episodes, respectively.

### Profile of Care-Seeking Patterns and Outcome Variables

Table 4 shows the profiles of the EDVS and the likelihood of subsequent events by patterns. Overall, the mean EDVS was 34.30. The NHC pattern exhibited a lower mean EDVS (32.77) than the overall mean. The HP pattern exhibited the highest mean (47.80). However, all of the ambulatory care patterns except for the RDLF pattern (30.51) exhibited high mean EDVS values.

**Table 4:** The profile of EDVS index and likelihood of ED subsequent events.

Pattern groups Care-seeking patterns	Mean of EDVS (Std)	Subsequent events	
		RED (%)	HA (%)
Total	34.30 (29.42)	8.93	22.02
Care-seeking patterns before ED visit			
Seeking directly ED care			
01: NHC→ED	32.77 (28.41)	7.22	15.55
To ED after hospitalization			
02: HP→ED	47.80 (33.92)	16.76	50.86
To ED after ambulatory care			
with RDSF (related-disease at same facility)			
***Subtotal***	***41*.*18*** ***(33*.*02)***	***11*.*51***	***36*.*01***
03: RDSF→ED	42.02 (33.29)	11.13	35.71
04: RDSF*2→ED	41.55 (33.00)	13.42	41.45
05: RDLF→RDSF→ED	37.40 (31.63)	11.35	32.31
with RDLF (related-disease at facility of lower or equal level)			
***Subtotal***	***30*.*51 (27*.*18)***	***9*.*27***	***18*.*69***
06: RDLF→ED	29.86 (26.77)	8.34	15.63
07: RDLF*2→ED	29.96 (26.91)	9.82	19.39
08: RDLF*3→ED	31.12 (27.49)	12.10	25.38
09: NDLF→RDLF→ED	34.69 (29.33)	10.81	28.44
with RDHF (related-disease at facility of higher level)			
10: RDHF→ED	36.22 (30.54)	13.19	26.22
with NDSF (non-related-disease at same facility)			
***Subtotal***	***40*.*93 (31*.*75)***	***11*.*47***	***38*.*15***
11: NDSF→ED	40.41 (31.67)	10.59	36.18
12: NDSF*2→ED	42.51 (31.92)	14.15	44.14
with NDLF (non-related-disease at facility of lower or equal level)			
***Subtotal***	***39*.*00 (31*.*36)***	***10*.*11***	***33*.*30***
13: NDLF→ED	38.20 (31.19)	9.17	29.70
14: NDLF*2→ED	41.40 (32.03)	10.66	40.80
15: NDLF*3→ED	43.57 (32.43)	13.06	46.22
16: RDLF→NDLF→ED	34.94 (29.29)	11.06	26.99
with NDHF (non-related-disease at facility of higher level)			
17: NDHF→ED	37.36 (30.17)	12.72	28.34

ED indicates emergency department; EDVS, the ED visit severity; RED, had returned to ED; HA, had hospital admission; NHC, no healthcare visit.

"99: XXXX*^n^" indicates the pattern 99 comprised n times consecutive visit of XXXX care type.

In the subsequent events, the overall RED rate was 8.93% and the HA rate was 22.02%. All of the patterns except for patterns containing NHC and RDLF visits exhibited RED and HA rates higher than the overall average. The HP pattern exhibited the highest RED (16.76%) and HA (50.86%) rates. In addition, more than one-third of the patterns including RDSF, NDSF, or NDLF were followed by a subsequent HA event, particularly those involving multi-NDSF/NDLF visits (> 40%).

### Adjusted Difference in EDVS and Subsequent Events

Table 5 shows the results obtained from the GENMOD model used to examine the difference in the EDVS and the likelihood of subsequent events among care-seeking patterns when we controlled for confounding variables. We observed that the EDVS was significantly associated with care-seeking patterns. Compared with NHC patterns, all other patterns except for those involving RDLF visits (the coefficients were -0.05) exhibited significantly higher EDVS values. Moreover, patterns containing single or multiple NDLF visits exhibited high EDVS values and an increase in the number of visits (the coefficients increased from 0.08 to 0.13).

**Table 5:** Adjusted association between Pre-ED care-seeking patterns and severity of ED visit, and subsequent events.


Pattern groups Care-seeking patterns	EDVS index ^a^ Paramater estimate β (95% CI)	Subsequent events after ED discharge	
		RED ^b^ Odds-Ratio (95% CI)	HA ^b^ Odds-Ratio (95% CI)
Care-seeking patterns before ED visit (RG=01: Seeking directly ED care)			
To ED after hospitalization			
02: HP→ED	0.13^‡^ (0.11,0.14)	1.86^‡^ (1.73,2.00)	2.32^‡^ (2.19,2.46)
To ED after ambulatory care			
with RDSF (related-disease at same facility)			
03: RDSF→ED	0.12^‡^ (0.11,0.13)	1.35^‡^ (1.29,1.40)	1.88^‡^ (1.82,1.94)
04: RDSF*2→ED	0.07^‡^ (0.05,0.09)	1.53^‡^ (1.42,1.65)	2.11^‡^ (1.98,2.25)
05: RDLF→RDSF→ED	0.06^‡^ (0.05,0.08)	1.52^‡^ (1.41,1.64)	2.20^‡^ (2.07,2.33)
with RDLF (related-disease at facility of lower or equal level)			
06: RDLF→ED	-0.05^‡^ (-0.06,-0.05)	1.21^‡^ (1.18,1.25)	1.16^‡^ (1.14,1.19)
07: RDLF*2→ED	-0.05^‡^ (-0.06,-0.04)	1.45^‡^ (1.40,1.51)	1.51^‡^ (1.46,1.55)
08: RDLF*3→ED	-0.05^‡^ (-0.06,-0.04)	1.77^‡^ (1.68,1.87)	1.96^‡^ (1.88,2.05)
09: NDLF→RDLF→ED	0.01 (-0.01,0.02)	1.46^‡^ (1.39,1.54)	1.89^‡^ (1.81,1.96)
with RDHF (related-disease at facility of higher level)			
10: RDHF→ED	0.05^‡^ (0.03,0.07)	1.58^‡^ (1.48,1.68)	1.64^‡^ (1.55,1.73)
with NDSF (with non-related-disease at same facility)			
11: NDSF→ED	0.03^‡^ (0.02,0.05)	1.19^‡^ (1.14,1.25)	1.69^‡^ (1.63,1.75)
12: NDSF*2→ED	0.02^†^ (0.01,0.04)	1.47^‡^ (1.36,1.60)	1.84^‡^ (1.74,1.95)
with NDLF (with non-related-disease at facility of lower or equal level)			
13: NDLF→ED	0.08^‡^ (0.07,0.08)	1.18^‡^ (1.14,1.22)	1.76^‡^ (1.72,1.81)
14: NDLF*2→ED	0.12^‡^ (0.11,0.14)	1.32^‡^ (1.25,1.39)	2.56^‡^ (2.47,2.66)
15: NDLF*3→ED	0.13^‡^ (0.12,0.15)	1.59^‡^ (1.47,1.72)	2.84^‡^ (2.69,2.99)
16: RDLF→NDLF→ED	0.03^†^ (0.01,0.04)	1.50^‡^ (1.41,1.60)	1.71^‡^ (1.63,1.80)
with NDHF (with non-related-disease at facility of higher level)			
17: NDHF→ED	0.02+ (0.00,0.03)	1.40^‡^ (1.32,1.49)	1.46^‡^ (1.39,1.53)

ED indicates emergency department; EDVS, ED visit severity; RED, had returned to ED; HA, had hospital admission.

^a^ Using generalized linear models with generalized estimating equation method based on gamma distribution with logarithmic link.

^b^ Using generalized linear models with generalized estimating equation mothed based on binominal distribution with logit link.

"99: XXXX*^n^" indicates the pattern 99 comprised n times consecutive visit of XXXX care type.

95% CI indicates 95% confidence interval. RG denotes reference group. "‡" indicates that p-value ≦ 0.001; "†" p-value ≦ 0.01; "+" p-value ≦ 0.05;

In subsequent events, the pattern of NHC care exhibited a significantly lower likelihood of involving a RED and HA than did other patterns. Compare with NHC pattern, the odds ratios ranged from 1.18 to 1.86 and 1.16 to 2.84. The HP pattern was the most likely to be followed by a RED (odds ratio = 1.86) and multi-NDLF patterns were the most likely to be followed by HA (odds ratio ≥ 2.56). Patterns involving ambulatory care at the same facility or at a facility of an equal or lower level were much more likely to be followed by a RED or HA as the number of visits of the same type increased.

## Discussion

This study was conducted to determine the specific pre-ED care-seeking patterns in a timeframe episode and to examine the association of these patterns with the EDVS and subsequent events. The results showed that up to 55% of ED visitors sought other type of care prior to ED visit. The most common pre-ED episode was ambulatory care for an RDLF associated with a low EDVS, followed by NDLF patterns associated with a high EDVS. The likelihood of subsequent events varied among patterns, and pattern NHC pattern was the least likely to be followed by subsequent events. In particular, among patterns with ambulatory care at the same facility or a facility of a lower or equal level, the likelihood that an ED visit was followed by subsequent events increased with the number of pre-ED visits.

A previous study has reported that a considerable percentage of post-ED HP are actually readmissions [43]. We observed that patients who visited an ED were associated with a higher likelihood of subsequent HA, had the highest EDVS, and were most likely to experience RED. This might partially be due to the discontinuity of post-discharge care, causing the patients to seek care from the ED and through readmission. An interventional study reported that intensive follow-up services provided through integrated post-discharge transition care can successfully reduce the readmission rate [44]. This finding suggests that, if hospitals actively scheduled continual follow-up care for their discharged patients, then the number of ED visits may be reduced as well.

In health policy, seeking care for nonurgent conditions has been considered synonymous with “inappropriate ED use” [3], and thus, care for nonurgent conditions has become the target of intervention programs designed to reduce ED crowding. From this perspective, previous studies have reported that more than half of ED visit were nonurgent visit in Taiwan [1,13]. Our study further showed that around 45% pre-ED care-seeking patterns were seeking ED care directly, and there is more likely to be lower EDVS and lowest subsequent HA and RED. This finding indicated that pattern seeking ED care directly may represent a substantial numbers of inappropriate utilization of ED resources and could be driven to treat at primary care. They could be a target population for designing effective strategy to reduce nonurgent ED visit.

Furthermore, pre-ED care-seeking patterns involving ambulatory care at a facility of a lower or equal level are most likely due to the unmet treatment needs, which could be a potential area for intervention. We observed that RDLF patterns exhibited a relatively low EDVS, whereas NDLF patterns were associated with high EDVS values. Moreover, RDLF and NDLF patterns were both associated with a high likelihood of subsequent events. These results indicates that, in addition to unmet service needs, patients sought ED care because of previous treatment may be inappropriate. Numerous studies have reported that frequent ED users also use other healthcare services heavily before visiting an ED [17–19]. Researchers have indicated that the characteristics and quality of primary care can influence ED use [4,7,45]. Our study provides further evidence supporting this finding. In a pre-ED timeframe episode, a certain number of ED users used ambulatory care more than once prior to ED visit. And the frequency of pre-ED visits was associated with an increased risk of subsequent events, even when we controlled for the EDVS. These results suggest that, in addition to increasing accessibility, an intervention policy focused on improving the capacity and quality of primary care may increase appropriate ED use. In addition, previous study has revealed that a large number of patients do not know what services can be offered by the office setting of their primary care physicians [46]. Our study also suggest that strategies to enhance primary care providers play a role in educating patients about services offered in the office setting and timely informed them of which medical condition would require care in the ED may reduce inappropriate ED use.

We also observed that patients who exhibited the RDSF patterns used ED services for urgent conditions. The possible reason that these patients used ED services appropriately is that, at the same facility, unmet needs caused by gap in treatment capacity are not a concern, and coordination among service departments enhances service. However, this pre-ED care-seeking pattern exhibited a high probability of subsequent RED and HA. Possibly because patient needs could not be met fully or in a timely manner, and barriers prevented timely HA. This result confirms the results of previous studies and suggests that enhanced coordination among hospital department can facilitate timely satisfaction of patient needs and, thus, reduce ED use by patients who exhibit the RDSF pattern.

This study had 4 limitations. First, we identified factors influencing the use of ED services based on the associations among sequential patterns of pre-ED health care seeking behaviors, ED use, and subsequent events. These associations cannot reflect the relationship between causes and effects, therefore, the application of our results limited. In addition, care-seeking patterns must be evaluated and adjusted according to the differences in health care systems. Second, we used the ICD-9-CM code group based on 3x3 diagnoses matching to categorize the pre-ED patterns into related or non-related disease. However, all the diagnostic codes of ED visitors were an impression for these patients that had been decided based on the ED physicians' experiences and all the medical evidences obtained during a limited period. Clinically, some ill-differentiated medical problems of the patients may change in diagnoses, especially in symptoms, signs, ill-defined condition (code in 780–799) and other nonspecific disease. Therefore, the changed coding may have limitation on false categorization bias. Third, our study used the NYU ED algorithm to categorize the diagnosis severity of ED visits based on the diagnosis code. Deficiencies in claims data involving missing coding information or coding errors may have contributed to under- or over-categorization. A study reported that the NYU ED algorithm insufficiently sensitive to changes in ED use patterns [47], potentially limiting the implications drawn from the study results. Fourth, the health care system in which Taiwan’s NHI operates does not emphasize a referral mechanism or promote the notion of having a family physician. Beneficiaries may exercise their freedom to choose any available provider for health care needs [48]. The care-seeking behaviors of patients in our study may differ considerably from those of patients in other countries. Therefore, caution should be exercised when generalizing our study results.

In conclusion, this study provides a foundation for investigating pre-ED care-seeking behaviors based on diverse care types and their association with severity of presenting condition for ED visit and subsequent events. The results suggest that pre-ED care-seeking patterns differ in severity of presenting condition for ED visit and subsequent events that may represent different causes of ED visit. Care-seeking behavior profiles can provide policy makers with useful information regarding how to differentiate target populations with different patterns, and further to create appropriate and different strategies to reduce unnecessary ED visit effectively.
